# Inoculation with *Azorhizobium caulinodans* ORS571 enhances plant growth and salt tolerance of switchgrass (*Panicum virgatum* L.) seedlings

**DOI:** 10.1186/s13068-023-02286-3

**Published:** 2023-03-02

**Authors:** Pengyang Chen, Qiannan Wei, Yifei Yao, Jiaqi Wei, Li Qiu, Baohong Zhang, Huawei Liu

**Affiliations:** 1 College of Life Sciences, Northwest A & F University, Yangling, 712100 Shaanxi China; 2 College of Veterinary Medicine, Northwest A & F University, Yangling, 712100 Shaanxi China; 3 Department of Biology, East Carolina University, Greenville, NC 27858 USA

**Keywords:** Switchgrass, Beneficial microorganism, Biofuel, MicroRNA, Salinity stress

## Abstract

**Background:**

Switchgrass (*Panicum virgatum* L.) is an important biofuel crop that may contribute to replacing petroleum fuels. However, slow seedling growth and soil salinization affect the growth and development of switchgrass. An increasing number of studies have shown that beneficial microorganisms promote plant growth and increase tolerance to salinity stress. However, the feasibility of inoculating switchgrass with *Azorhizobium caulinodans* ORS571 to enhance the growth and salt tolerance of its seedlings is unclear. Our previous study showed that *A*. *caulinodans* ORS571 could colonize wheat (*Triticum aestivum* L.) and thereby promote its growth and development and regulate the gene expression levels of microRNAs (miRNAs).

**Results:**

In this study, we systematically studied the impact of *A*. *caulinodans* ORS571 on switchgrass growth and development and the response to salinity stress; we also studied the underlying mechanisms during these biological processes. Inoculation with *A*. *caulinodans* ORS571 significantly alleviated the effect of salt stress on seedling growth. Under normal conditions, *A*. *caulinodans* ORS571 significantly increased fresh plant weight, chlorophyll a content, protein content, and peroxidase (POD) activity in switchgrass seedlings. Under salt stress, the fresh weight, dry weight, shoot and root lengths, and chlorophyll contents were all significantly increased, and some of these parameters even recovered to normal levels after inoculation with *A*. *caulinodans* ORS571. Soluble sugar and protein contents and POD and superoxide dismutase (SOD) activities were also significantly increased, contrary to the results for proline. Additionally, *A*. *caulinodans* ORS571 may alleviate salt stress by regulating miRNAs. Twelve selected miRNAs were all upregulated to different degrees under salt stress in switchgrass seedlings. However, the levels of miR169, miR171, miR319, miR393, miR535, and miR854 were decreased significantly after inoculation with *A*. *caulinodans* ORS571 under salt stress, in contrast to the expression level of miR399.

**Conclusion:**

This study revealed that *A*. *caulinodans* ORS571 increased the salt tolerance of switchgrass seedlings by increasing their water content, photosynthetic efficiency, osmotic pressure maintenance, and reactive oxygen species (ROS) scavenging abilities and regulating miRNA expression. This work provides a new, creative idea for improving the salt tolerance of switchgrass seedlings.

**Supplementary Information:**

The online version contains supplementary material available at 10.1186/s13068-023-02286-3.

## Background

Switchgrass is a perennial lignocellulose biofuel crop that has been developed as an important bioenergy feedstock due to its high production and viability [1, 2]. By analysing the biomass yield and agricultural input model of switchgrass, it has been found that the energy output of switchgrass is much higher than the energy input it receives, which makes switchgrass an excellent alternative candidate for replacing petroleum fossil fuels [3–5]. However, the slow growth rate of seedlings makes switchgrass less competitive with weeds, which significantly affects the utilization of switchgrass [3]. Improving the competitiveness of seedlings for survival and optimizing the growth of switchgrass are of great significance for increasing its yield and reducing the cost of planting [6, 7].

Soil salinization is one of the major abiotic stresses affecting global agricultural production, and approximately 20% of the world's farmland is severely affected by salt stress [8, 9]. High Na^+^ concentrations in soil lead to hyperosmotic and hyperionic conditions, which further inhibit plant nutrient intake [10]. A high level of sodium toxicity causes osmotic stress and ionic toxicity in plants and increases oxidative stress in particular. Salt stress induces plants to produce reactive oxygen species (ROS), mainly in the form of singlet oxygen (^1^O_2_), superoxide anion (O_2_^−^), and hydrogen peroxide (H_2_O_2_). These ROS attack lipids and proteins, which causes cell damage and death [11–14]. A series of enzymatic antioxidant defence systems exist in plant cells to remove excessive ROS, including peroxidase (POD) and superoxide dismutase (SOD) [15, 16]. In addition, the levels of various soluble osmotic substances are greatly increased under salt stress, which is extremely important for regulating osmotic pressure in plants [9]. Salt stress also severely affects the biosynthesis of chlorophyll, which in turn affects photosynthesis [17]. Thus, salt stress affects various aspects of plant physiology and anabolic metabolism. MicroRNAs (miRNAs) are a very large class of endogenous small RNAs that are involved in regulating plant development and responses to biotic or abiotic stresses by negatively regulating the expression of their target genes at the mRNA level [18–20]. The main abiotic stresses that plants must face include salt, drought, cold, and heat stress. A series of miRNAs responding to salt stress have been identified, and the discovery of these miRNAs greatly enriched the known salt stress regulatory network of plants [21].

*A. caulinodans* ORS571 was originally isolated from stem nodules of the tropical leguminous plant *Sesbania rostrata* and has the ability to nodulate both roots and stems of the host plant [22, 23]. Interestingly, our previous work showed that *A. caulinodans* ORS571 could colonize wheat and promote the growth of its leaves, roots, and biomass [22, 24]. In recent years, researchers have demonstrated that the colonization of certain microorganisms is beneficial to plant development and growth. The strain *Raoultella terrigena* R1Gly, which was isolated from tobacco, can colonize switchgrass and promote the development of roots, and *Enterobacter sp.* SA187 and *Piriformospora indica* increase the salt tolerance of *Arabidopsis* and tomato, respectively [25–28]. However, it is unclear whether *A. caulinodans* ORS571 affects the growth and salt resistance of switchgrass.

This study provided evidence regarding the colonization of switchgrass by *A. caulinodans* ORS571 and its effects on switchgrass plant growth and development. The changes in physiological indexes and miRNA expression in switchgrass seedlings were also discussed. This will provide a strong basis for promoting switchgrass production and biofuel development.

## Methods and materials

### Plant growth and inoculation

Switchgrass Alamo seeds were kindly provided by Dr. Neal Stewart at the University of Tennessee at Knoxville. Switchgrass seeds were planted in the Plant Growth Room under 16 h of light at a constant temperature of 28 ℃ and 8 h of dark at 18 ℃. *gfp*-*A. caulinodans* ORS571 was kindly provided by Professor Yuxiang Jing of the Institute of Botany, Chinese Academy of Sciences [29]. *A. caulinodans* ORS571 was cultured in tryptone yeast (TY) liquid medium at 28 ℃ and 140 rpm until the OD_600_ reached 0.6 and then suspended in sterile water to 10^8^ cells per millilitre [22].

The switchgrass seeds used in this experiment were surface sterilized with 70% ethanol for 90 s and 5% sodium hypochlorite (NaClO) for 10 min, followed by rinsing several times with sterile distilled water [22]. Sterilized seeds were placed in 15-cm-diameter pots filled with sterile vermiculite for germination. Three biological replicates were set up for each experimental group, and 50 seeds were germinated for each replicate. The pots were irrigated with 1/4 strength Hoagland solution (pH 6.5) before planting. After switchgrass germination, 0.5% NaCl was applied for 5 days, followed by 1% NaCl for 10 days [25]. Meanwhile, a 50 mL aliquot of the bacterial solution was inoculated into the root zone at 1 and 6 days. Additionally, sterile water was applied to the controls.

### Observation of A. caulinodans ORS571 in root tissue

Two weeks after the treatments, the roots of switchgrass seedlings were observed by laser confocal microscopy (Leica, Bensheim, Germany) at a wavelength of 488 nm to monitor *A. caulinodans* ORS571 colonization.

### Plant growth parameters

The lengths of the shoots and roots and the total biomass of switchgrass seedlings were determined after 15 days of salt stress treatment. The fresh weight and dry weight of the seedlings were measured. The dry weight was measured by placing the washed seedlings in an oven at 80 °C until the weight remained constant [30].

### Measurement of chlorophyll and proline contents

Chlorophyll was extracted by using ethanol as the extraction solvent. Switchgrass leaf samples of approximately 0.5 g were washed with distilled water, cut into pieces, added to an appropriate amount of quartz sand and calcium carbonate, and ground in 95% ethanol. After filtering, the grinding liquid was diluted to 25 mL with 95% ethanol and then subjected to measurement in a spectrophotometer at 665 nm and 649 nm [31].

Approximately 0.5 g of switchgrass leaf extract was added to 5 mL of 3% sulfosalicylic acid, and the mixture was placed in a water bath at 100 °C for 10 min. Equal volumes of glacial acetic acid and acidic ninhydrin solution were added to 2 mL of the extract, which was then placed in boiling water for 30 min until the solution turned red. Then, 4 mL of toluene was added, and the mixture was shaken for 30 s and centrifuged at 3000 rpm for 5 min. Finally, the upper liquid layer was collected, and the proline content was measured at a wavelength of 520 nm [32].

### Determination of soluble sugar and soluble protein contents

The soluble sugar content was measured via the anthrone colorimetric method. Approximately 0.5 g of fresh leaf was added to 10 mL of distilled water, and this mixture was then placed in boiling water for 30 min and filtered into a 25 mL volumetric flask after cooling. Thereafter, a mixture of 1.5 mL distilled water, 0.5 mL anthrone ethyl acetate, 0.5 mL sample, and 5 mL concentrated sulfuric acid was prepared, shaken vigorously for 30 s, incubated in boiling water for 10 min, and subjected to measurement at a wavelength of 620 nm after cooling down [33].

The Coomassie brilliant blue method was used for the determination of soluble protein content. Approximately 0.5 g of fresh leaf was ground to powder with distilled water, centrifuged to collect the supernatant, and then diluted to 10 mL. Thereafter, 5 mL of Coomassie brilliant blue was mixed with 0.1 mL of the sample, and the mixture was shaken vigorously for 2 min and measured at a 595 nm wavelength [34].

### Measurement of antioxidant enzyme activities

Approximately 0.2 g of switchgrass leaf was ground to a powder in 5 mL of potassium phosphate buffer (50 mM, pH 7.8) containing 1% PVP and then centrifuged at 12,000 × g for 10 min at 4 °C. The supernatant was collected for subsequent measurements. The enzymatic activity of SOD was determined according to its ability to inhibit O_2_^−^-induced NBT reduction [35]. The activity of POD was assessed according to the rate at which guaiacol was oxidized by hydrogen peroxide, and enzyme activity was calculated by recording the absorbance change at 470 nm [36].

### RNA extraction and quantitative real-time PCR (qRT-PCR)

Three switchgrass seedlings from each pots were selected, washed, chopped, and mixed, and the samples were then weighed. Total RNA was extracted by using TRIzol^®^ Reagent (Invitrogen, Carlsbad, CA, USA) according to the manufacturer’s instructions. The quality of the extracted total RNA was checked by Nanodrop ND-1000 and gel electrophoresis analyses. Reverse transcription (RT) reactions were implemented with a One Step PrimeScript^®^ miRNA cDNA synthesis kit (TaKaRa, Dalian, China). qRT-PCR was run on a BIO-RAD CFX96TM system, and three biological replicates were performed for each miRNA. The qRT-PCR results were analysed by using the 2^−ΔΔCT^ method [37].

### Statistical analysis

All experiments were replicated three times, and the results are presented as the mean ± SD (standard deviation). One-way ANOVA was employed to analyse the significant differences (*P* < 0.05) among different treatments. The same letters indicate that the results were not significantly different. Student’s *t-test* was used for the statistical analysis of qRT-PCR results (*P < 0.05, **P < 0.01). Both analyses were performed in GraphPad Prism 8.0.2 software (*n* ≥ 3).

## Results

### A. caulinodans ORS571 alleviated salt stress in switchgrass seedlings

**Fig. 1:**
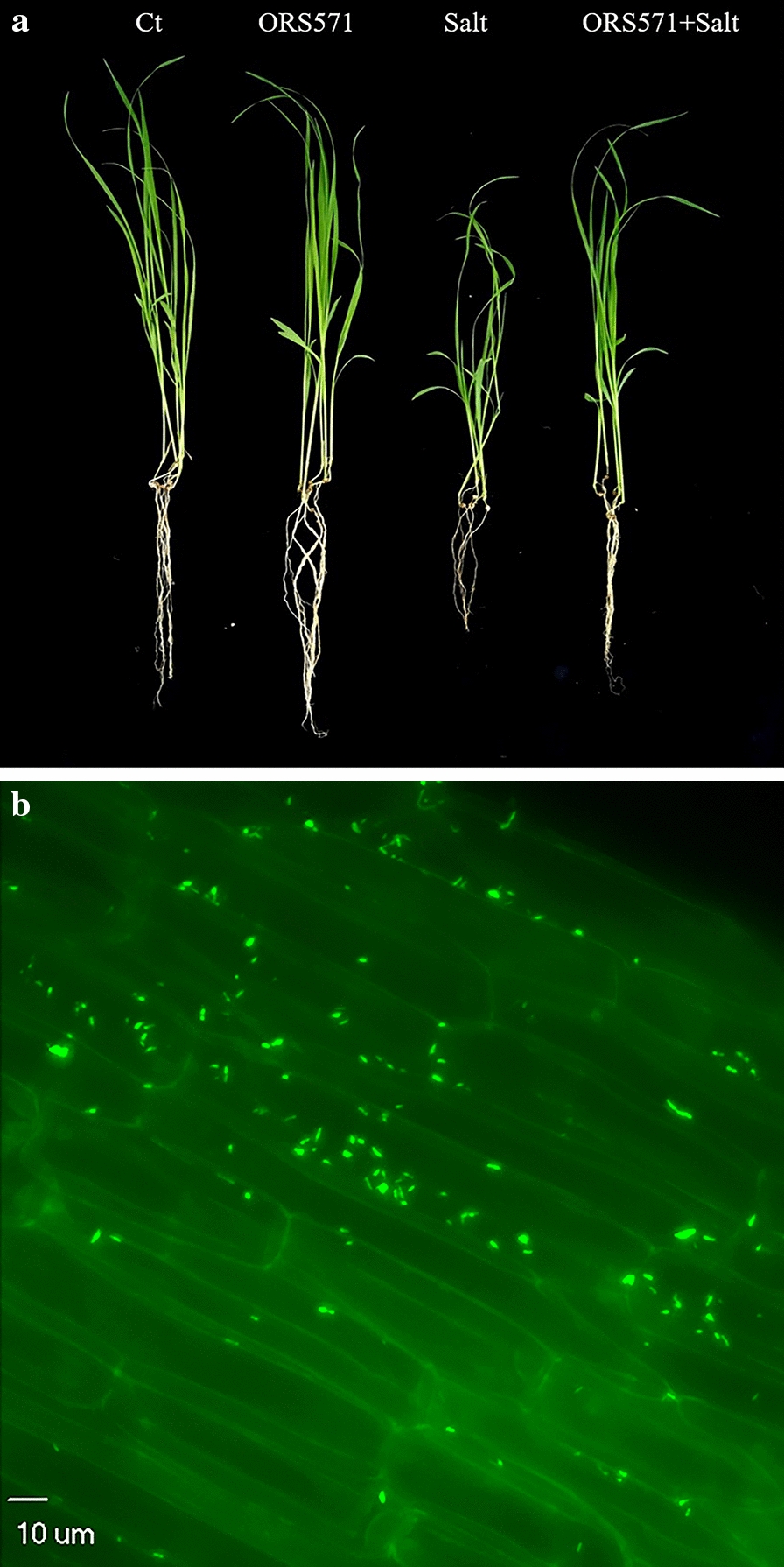
Colonization of *A. Caulinodans* ORS571 in switchgrass. **a** Effects of *A. Caulinodans* ORS571 on switchgrass seedlings under normal and salt stress; “Ct” means control group without any treatment; **b** The colonization of *A. Caulinodans* ORS571 in the roots of switchgrass seedlings

**Fig. 2:**
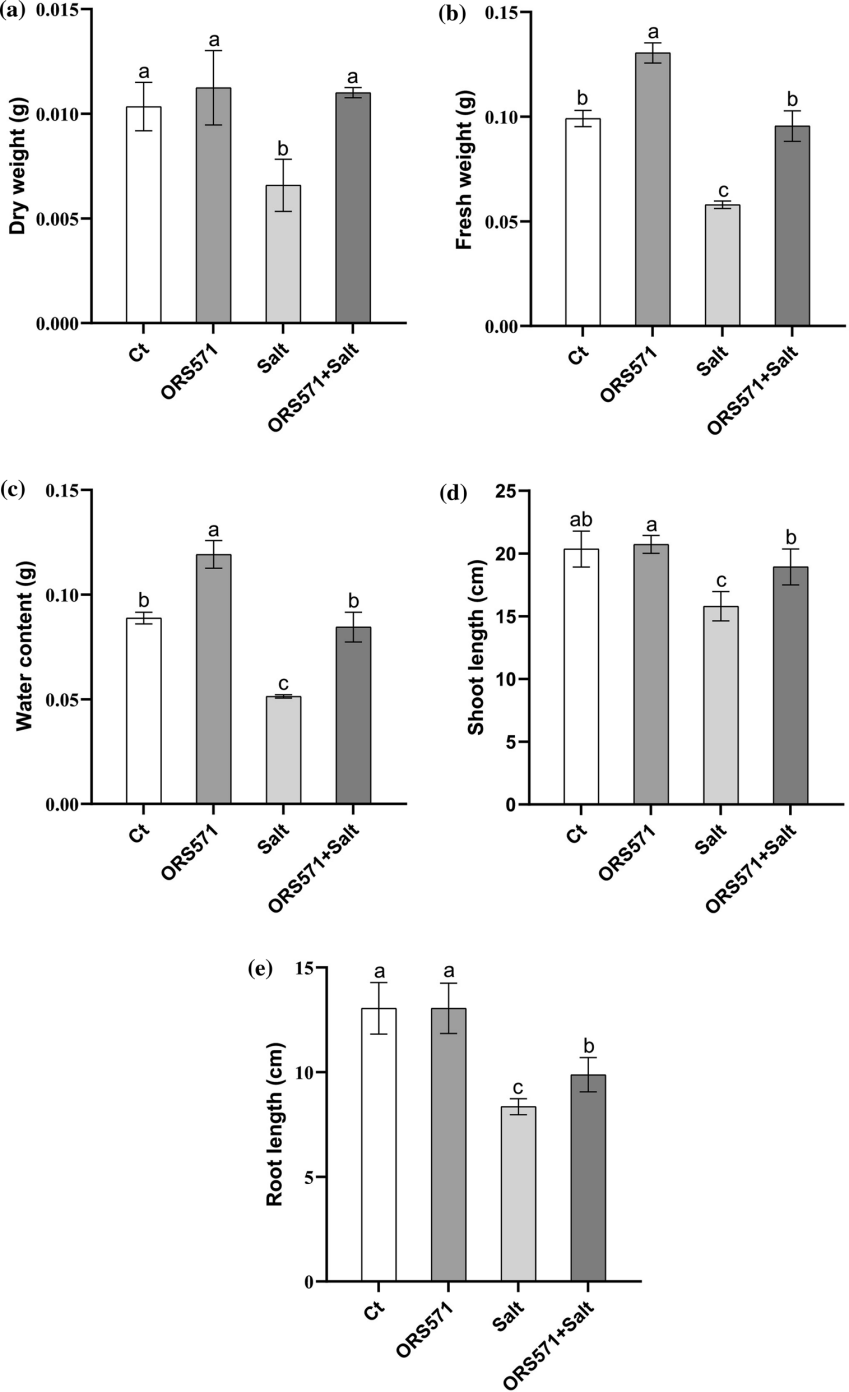
Effects of *A. Caulinodans* ORS571 on the growth status of switchgrass seedlings. **a** Dry weight; **b** Fresh weight; **c** Water content; **d** Shoot length; **e** Root length. The same letters indicate that the result is not significant (*P* < 0.05) according to one-way ANOVA; error bars are standard error (SE) (dry weight, fresh weight, and water content, *n* = 3; shoot length and root length, *n* = 10)

To explore the effect of salt stress on seedling development, different salt concentrations (0%, 0.5%, 1%, and 1.5%) were applied in a preliminary experiment. The results showed that 1% NaCl was sufficient to cause significant stress to seedlings (Additional file 1: Figure S1), while the presence of *A. caulinodans* ORS571 could alleviate the damage caused by salt stress to varying degrees (Fig. 1a; Additional file 1: Figure S2). Two weeks after inoculation, *A. caulinodans* ORS571 was observed in the roots of switchgrass seedlings (Fig. 1b). Under salt stress treatments, the colonization of *A*. *caulinodans* ORS571 resulted in significant increases in the total fresh weight, dry weight, and shoot and root lengths of switchgrass seedlings (Fig. 2). The biomass of seedlings inoculated with *A. caulinodans* ORS571 was increased by 8.7% and 67.2% under normal and salt stress conditions, respectively, and the fresh weight and water content were also increased (Fig. 2a–c). Salt stress inhibited both shoot and root development, while *A*. *caulinodans* ORS571 inoculation significantly increased shoot and root development by 19.8% and 18.3%, respectively (Fig. 2d, e). The colonization of *A*. *caulinodans* ORS571 even caused the fresh weight, dry weight, and shoot length of seedlings to recover to nearly normal levels.

### A. caulinodans ORS571 significantly increased the contents of chlorophyll, soluble sugar, and protein in switchgrass seedlings under salt stress

**Fig. 3:**
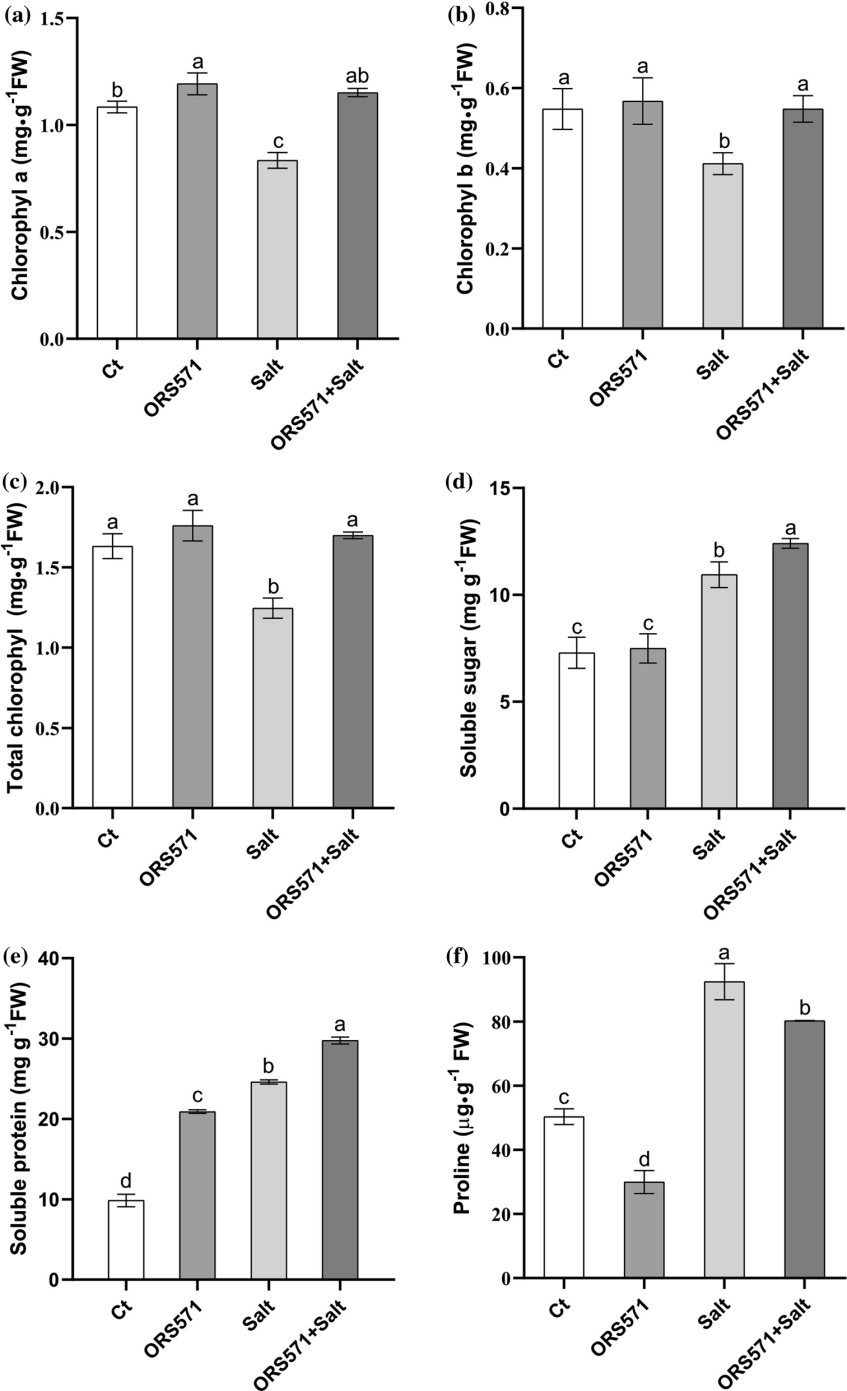
*A. caulinodans* ORS571 promoted the contents of chlorophyll and soluble solids and decreased proline content under salt stress. **a** Chlorophyll a content; **b** Chlorophyll b content; **c** Total chlorophyll content; **d** Soluble sugar content; **e** Soluble protein content; **f** Proline content. The same letters indicate that the result is not significant (*P* < 0.05) according to one-way ANOVA; error bars are standard error (SE), *n* = 3

Significant increases in the content of chlorophyll a by 9.9% and 38.1% were observed in inoculated seedlings under both normal and salt stress conditions, respectively (Fig. 3a). Although the chlorophyll b content of inoculated seedlings was also significantly increased by 33.2% under salt stress, it was almost unchanged under normal conditions (Fig. 3b). The change in the total chlorophyll contents was consistent with the change in the chlorophyll b content (Fig. 3c). Salt stress significantly increased the contents of soluble sugar and soluble protein by 50.2% and 149.6%, respectively, which were further significantly increased after inoculation with *A*. *caulinodans* ORS571 (Fig. 3d, e). In addition, inoculation with *A*. *caulinodans* ORS571 under normal conditions resulted in a significant increase in the soluble protein content (Fig. 3e). Proline showed abundant accumulation under salt stress. However, it was noteworthy that the contents of proline were significantly decreased by 40.5% and 13.2% after inoculation under normal and salt stress conditions, respectively (Fig. 3f).

### A. caulinodans ORS571 significantly increased POD and SOD activities in switchgrass seedlings under salt stress

**Fig. 4:**
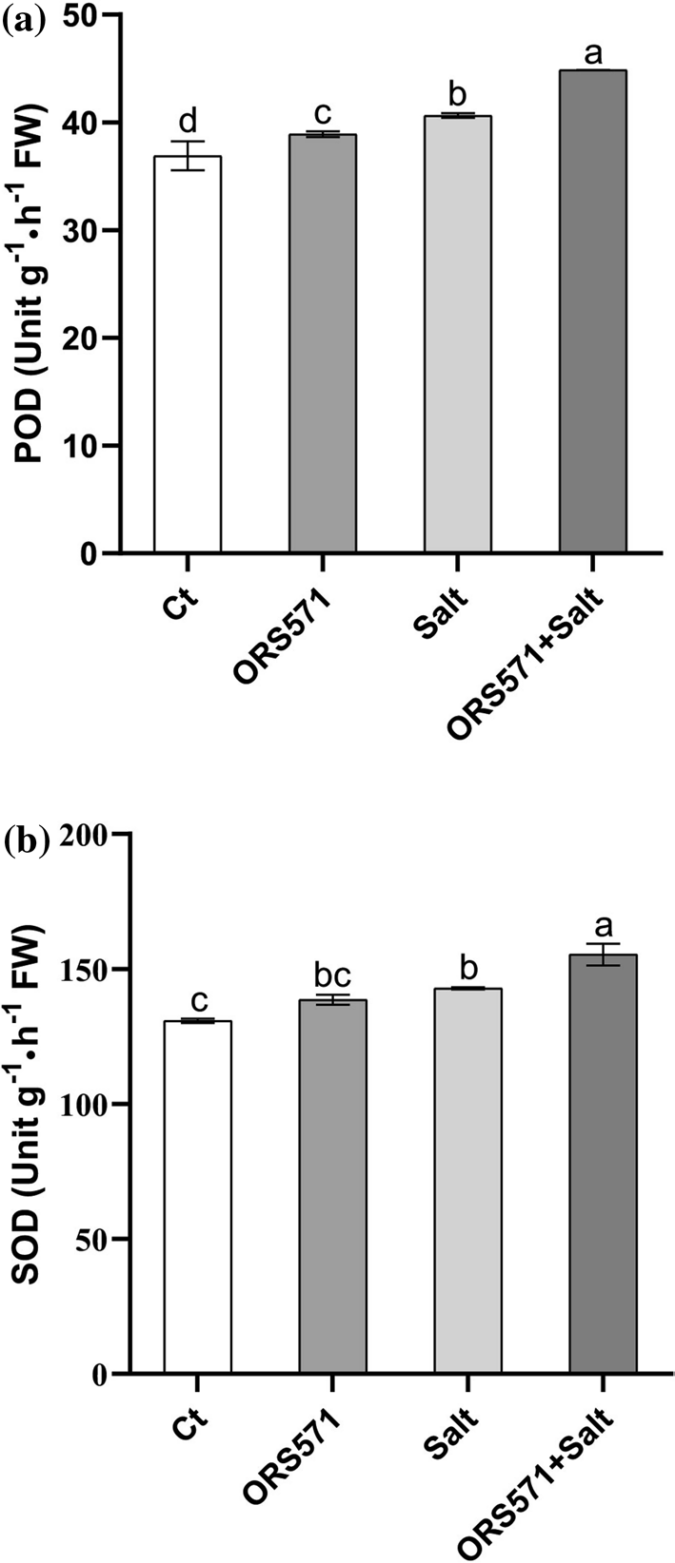
*A. caulinodans* ORS571 improved activities of POD and SOD. **a** Activity of POD; **b** Activity of SOD. The same letters indicate that the result is not significant (*P* < 0.05) according to one-way ANOVA; error bars are standard error (SE), *n* = 3

POD and SOD are the main enzyme components of plant cells that defend against oxidative damage. The activities of POD and SOD were significantly increased under salt stress, which were further increased by 10.3% and 8.8%, respectively, in *A. caulinodans* ORS571-colonized plants (Fig. 4). There was a significant increase in the activity of POD after inoculation with *A. caulinodans* ORS571 under normal conditions, but SOD activity was not affected.

### A. caulinodans ORS571 affected the differential expression of miRNAs in switchgrass seedlings under salt stress

**Fig. 5:**
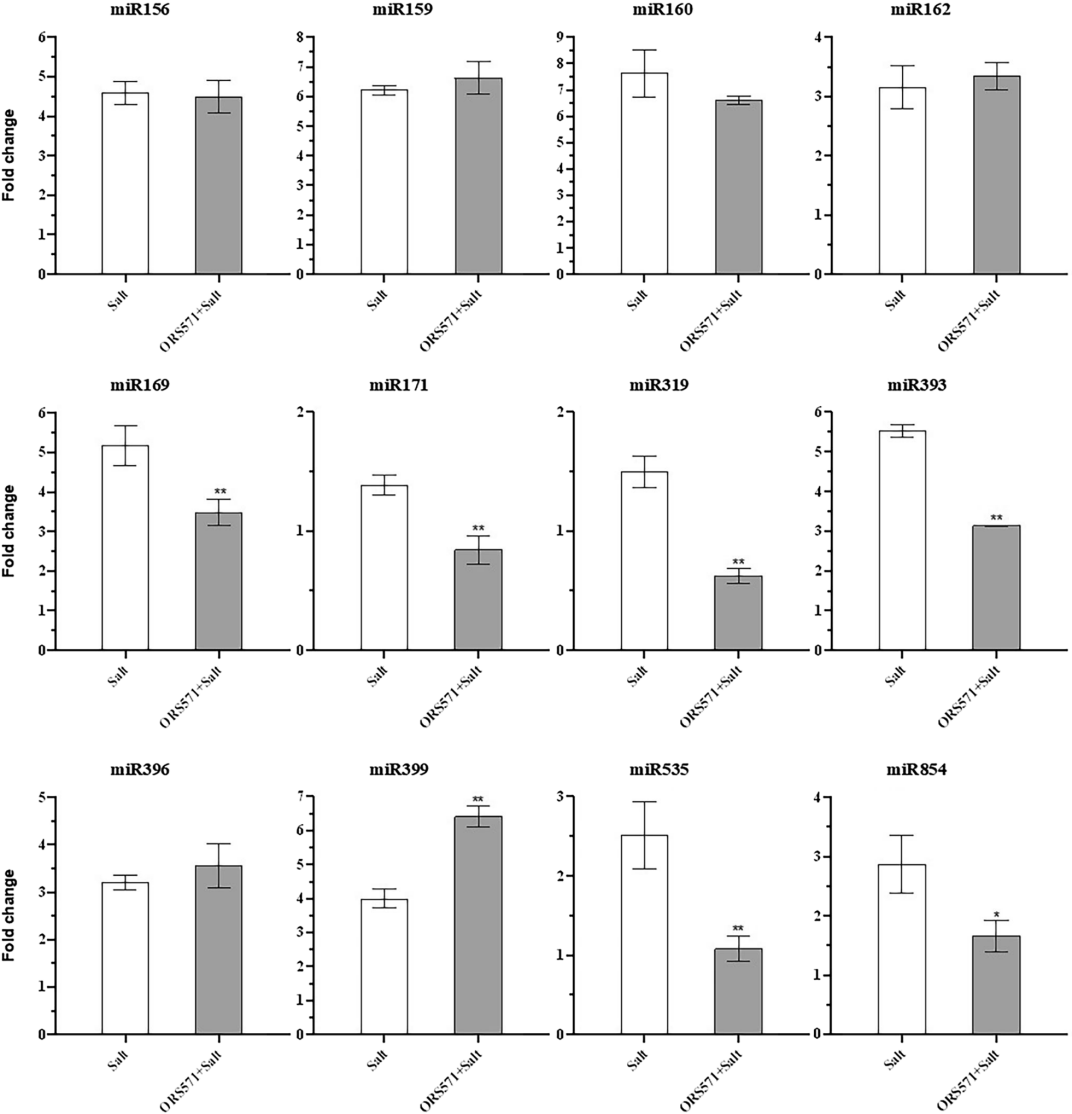
Differential gene expression level of miRNAs. Significance according to Student’s* t*-test results (*P < 0.05, **P < 0.01). Error bars are standard error (SE), *n* = 3. Fold change means the change of gene expression level of miRNAs compared to “Ct” group

To study the role of miRNAs in the switchgrass response to *A. caulinodans* ORS571 and the potential mechanism underlying the increased plant growth and tolerance of *A. caulinodans* ORS571-infected switchgrass to abiotic stresses, we evaluated the expression of miRNAs associated with plant growth and stress responses. In this study, twelve miRNAs were selected, and we found that all miRNAs were upregulated under salt stress and that miR399 expression was further increased after inoculation with *A. caulinodans* ORS571 (Fig. 5). The expression levels of miR169, miR171, miR319, miR393, miR535, and miR854 were significantly decreased after treatment with bacteria, and the expression trends of miR171 and miR319 were even downregulated. However, the expression levels of miR156, miR159, miR160, miR162, and miR396 did not change significantly.

## Discussion

### A. caulinodans ORS571 significantly increased switchgrass seedling biomass under salt stress

There is growing evidence that beneficial microorganisms enhance plant tolerance to various environmental stresses [25, 38, 39]. There are seldom studies on the colonization of plants by *A. caulinodans* ORS571 and its promotion of plant growth and development, although our previous study demonstrated the colonization of *A. caulinodans* ORS571 in the roots and leaves of wheat [22, 24]. We further identified the colonization of the roots of switchgrass seedlings by this strain and its positive effect on improving salt tolerance in the present study (Fig. 1b). Salt stress adversely affected the growth and development of switchgrass seedlings, and these effects were significantly alleviated by inoculation with *A. caulinodans* ORS571 (Fig. 1a), resulting in significant increases in the shoot and root lengths and dry and fresh weights of switchgrass (Fig. 2). Additionally, all of these parameters except root length recovered to nearly normal levels after *A. caulinodans* ORS571 treatment. Contrary to the results of previous studies in wheat, the biomass and root and shoot lengths of switchgrass seedlings did not change after inoculation with *A*. *caulinodans* ORS571 under normal conditions, but their water content was significantly increased (Fig. 2c) [24]. These differences may be due to the slow growth of switchgrass at the seedling stage [3]. The significant increase in water content means that the water retention capacity of switchgrass is increased, which has a positive effect on promoting seedling growth and salinity tolerance [40]. The changes in these growth parameters reflected the positive effect of *A. caulinodans* ORS571 on the enhancement of salt tolerance in switchgrass seedlings.

Salt stress significantly reduced the contents of chlorophyll a and chlorophyll b in switchgrass seedlings, which were significantly increased after inoculation with *A*. *caulinodans* ORS571 (Fig. 3a–c). Chlorophyll content directly determines the photosynthetic efficiency of plants, which is positively correlated with biomass and plant salt tolerance [41]. The observed alleviation of the damage to chlorophyll indicated that *A*. *caulinodans* ORS571 increased the tolerance of switchgrass seedlings to salt stress. However, the total chlorophyll contents under normal conditions was not affected by *A*. *caulinodans* ORS571, which was consistent with the results for other growth parameters measured in our experiment, suggesting that *A*. *caulinodans* ORS571 may only respond specifically to certain stresses in switchgrass seedlings.

### A. caulinodans ORS571 significantly increased soluble sugar/protein contents and decreased proline content in switchgrass seedlings under salt stress

Soluble sugar, which is the main product of photosynthesis and the substrate of respiration, plays a critical role in plant growth and metabolism [42–44]. Soluble sugar is also used to maintain the osmotic homeostasis of cells and improve the stress resistance of plants [9, 45]. Soluble proteins usually accumulate under abiotic stresses. In addition to acting as osmotic regulators in plants, soluble proteins are crucial for the precise transmission of stress signals [46]. Consistent with previous research results, salt stress resulted in a significant increase in soluble sugar and protein contents in switchgrass [47], and the contents of soluble sugar and protein increased further after inoculation with *A*. *caulinodans* ORS571 (Fig. 3d, e). Under normal conditions, the content of soluble protein was significantly increased by *A*. *caulinodans* ORS571 inoculation, which suggests that this bacterium also plays a role in promoting growth by increasing the accumulation of amino acids.

Proline acts as a solute to maintain cellular osmotic balance and protect plant cells from various stresses [15, 48–50]. In this study, the proline content increased significantly under salt stress. Interestingly, inoculation with *A. caulinodans* ORS571 resulted in a significant decrease in proline content under both salt stress and normal conditions (Fig. 3f), which was different from the effects on soluble sugar and protein contents. Excessive free proline inhibits leaf development and even induces cell death in *Arabidopsis* [51, 52]. Therefore, *A. caulinodans* ORS571 may be beneficial for degrading excess proline in plant cells under normal conditions or when salt stress is alleviated.

### A. caulinodans ORS571 significantly induced the activities of antioxidant enzymes in switchgrass seedlings under salt stress

Under salt stress, plants produce and accumulate ROS, causing cell damage or death [11–14], and increases in POD and SOD activities are beneficial for scavenging excess ROS [53]. The activities of POD and SOD in *Zea mays*, *Arabidopsis,* and *Trichoderma longibrachiatum* are markedly increased under stress [54–56], consistent with our findings in switchgrass seedlings. Our study evaluated the activities of POD and SOD to explore whether inoculation with *A*. *caulinodans* ORS571 improved the tolerance of switchgrass seedlings to salt stress. The results showed that *A*. *caulinodans* ORS571 resulted in significant increases in POD and SOD activities under salt stress conditions, and POD activity was significantly increased even under normal conditions (Fig. 4a, b). These results suggest that the enhancement of ROS scavenging ability may be an important reason for the colonization of *A*. *caulinodans* ORS571 to alleviate salt toxicity and improve the salt tolerance of seedlings [39].

### Differential expression of miRNAs in switchgrass seedlings under salt stress

In previous studies, we identified a series of salt stress-related miRNAs in switchgrass by high-throughput, deep sequencing. Among the 12 miRNAs involved in this study (Table 1), 10 have been shown to respond to multiple abiotic stresses, including miR156, miR159, miR160, miR162, miR169, miR171, miR319, miR393, miR396, and miR399 [57–65]. miR535 and miR854 have been found to be associated with disease resistance in rice and essential oil synthesis in ginger, respectively [66, 67]. Our results showed that the twelve miRNAs were all upregulated to varying degrees under salt stress (Fig. 5). The changes in miR156, miR159, miR162, miR169, miR171, miR393, and miR396 were also consistent with our previous findings in switchgrass [63, 65]. The expression trend of miR160 was consistent with results obtained in peanut and the C4 plant sugarcane [68, 69], while it was opposite to our previous results [63], possibly because the concentration of salt used in the current study was twice that used previously. In previous studies, miR399 has been shown to be upregulated under low-concentration salt stress and slightly downregulated under high-concentration salt stress [65]. Perhaps because of the applied pretreatment, miR399 was still found to be upregulated under 1% NaCl treatment. miR319 was also upregulated, which was consistent with findings in creeping bentgrass [64], and the upregulation of miR535 and miR854 indicated that they may be also associated with salt stress.

**Table 1:** Primers of twelve miRNAs for qRT-PCR

Name of primers	Primer sequence (5′–3′)
miR156	UGACAGAAGAGAGUGAGCAC
miR159	UUUGGAUUGAAGGGAGCUCUA
miR160	UGCCUGGCUCCCUGUAUGCC
miR162	UCGAUAAACCUCUGCAUCCAG
miR169	CAGCCAAGGAUGACUUGCCGA
miR171	UUGAGCCGCGUCAAUAUCUCC
miR319	UUGGACUGAAGGGUGCUCCC
miR393	UCCAAAGGGAUCGCAUAUC
miR396	UCCACAGGCUUUCUUGAACUG
miR399	UGCCAAAGGAGAUUUGCCCUG
miR535	UGACAACGAGAGAGAGCACGC
miR854	GAUGAGGAUAGGGAGGAGGAG

Under salt stress, the expression of miR156, miR159, miR160, miR162, and miR396 was almost unchanged in switchgrass seedlings after inoculation with *A*. *caulinodans* ORS571, indicating that inoculation with *A*. *caulinodans* ORS571 did not affect the expression of these miRNAs or their expressions were relatively stable. The significant upregulation of miR399 indicated that inoculation with *A*. *caulinodans* ORS571 increased the salt tolerance of switchgrass seedlings. However, miR169, miR393, miR535, and miR854 levels were significantly decreased, probably because salt stress was alleviated. miR171 and miR319 are involved in legume nodulation and arbuscular mycorrhizal symbiosis, and the downregulation of miR171 and miR319 may be related to the *A*. *caulinodans* ORS571 colonization of switchgrass [70]. Therefore, *A*. *caulinodans* ORS571 increases the salt tolerance of switchgrass seedlings, possibly by regulating the expression of certain miRNAs. Overall, these results provide a foundation for studying the mechanism by which *A*. *caulinodans* ORS571 improves the salt tolerance of switchgrass.

## Conclusions

In this study, we investigated the effects of inoculation with *A*. *caulinodans* ORS571 by analysing growth parameters, physiological indicators, and miRNA expression patterns in switchgrass seedlings under normal and salt stress conditions. Under normal conditions, inoculation with *A*. *caulinodans* ORS571 significantly increased fresh weight, chlorophyll a content, protein content, and POD activity in switchgrass seedlings. Under salt stress, the fresh weight, dry weight, shoot and root lengths, and chlorophyll content were all significantly increased, and some of these parameters even recovered to normal levels after inoculation with *A*. *caulinodans* ORS571. The contents of soluble sugar and protein and POD and SOD activities were also significantly increased, in contrast to the findings for proline. It was suggested that inoculation with *A*. *caulinodans* ORS571 could enhance the salt tolerance of seedlings by increasing their water content, photosynthetic efficiency, osmotic pressure maintenance, and ROS scavenging abilities. Additionally, *A*. *caulinodans* ORS571 may alleviate salt stress by regulating miRNAs. Our results revealed that the inoculation of switchgrass with *A*. *caulinodans* ORS571 significantly improved salt tolerance and suggested an environmentally friendly solution for improving the competitiveness of seedlings and increasing biomass.

## Supplementary Information

**Additional file 1: Figure S1.** Effects of different NaCl concentrations on the growth status of switchgrass seedlings. a Effects of different NaCl concentrations on the phenotype of shoots; b Shoot length. The same letters indicate that the result is not significant (P<0.05) according to one-way ANOVA; error bars are standard error (SE), n = 10. **Figure S2.** *A. caulinodans* ORS571 and the switchgrass seedlings response to different concentrations of NaCl. The same letters indicate that the result is not significant (P<0.05) according to one-way ANOVA; error bars are standard error (SE), n = 10.

## Data Availability

The authors promise the availability of supporting data, and the data used and analysed during the current study are available from the corresponding author upon reasonable request.
