# Supplementary material for: Inoculation with Azorhizobium caulinodans ORS571 enhances plant growth and salt tolerance of switchgrass (Panicum virgatum L.) seedlings
Source: Biotechnol Biofuels Bioprod. 2023 Mar 2;16:35. doi: 10.1186/s13068-023-02286-3 (PMC9983177; doi:10.1186/s13068-023-02286-3)
Supplement: Supplementary file 1 — Additional file 1: Figure S1. Effects of different NaCl concentrations on the growth status of switchgrass seedlings. a Effects of different NaCl concentrations on the phenotype of shoots; b Shoot length. The same letters indicate that the result is not significant (P<0.05) according to one-way ANOVA; error bars are standard error (SE), n = 10. Figure S2. A. caulinodans ORS571 and the switchgrass seedlings response to different concentrations of NaCl. The same letters indicate that the result is not significant (P<0.05) according to one-way ANOVA; error bars are standard error (SE), n = 10. [file 13068_2023_2286_MOESM1_ESM.docx]

Additional file

**Figure S1.** Effects of different NaCl concentrations on the growth status of switchgrass seedlings.

**a** Effects of different NaCl concentrations on the phenotype of shoots;

**b** Shoot length. The same letters indicate that the result is not significant (*P<*0.05) according to One-way ANOVA, error bars are standard error (SE), n=10.

**Figure S2.** *A. caulinodans* ORS571 and the switchgrass seedlings response to different concentrations of NaCl. The same letters indicate that the result is not significant (*P<*0.05) according to One-way ANOVA, error bars are standard error (SE), n=10.

**Figure S1a**

**
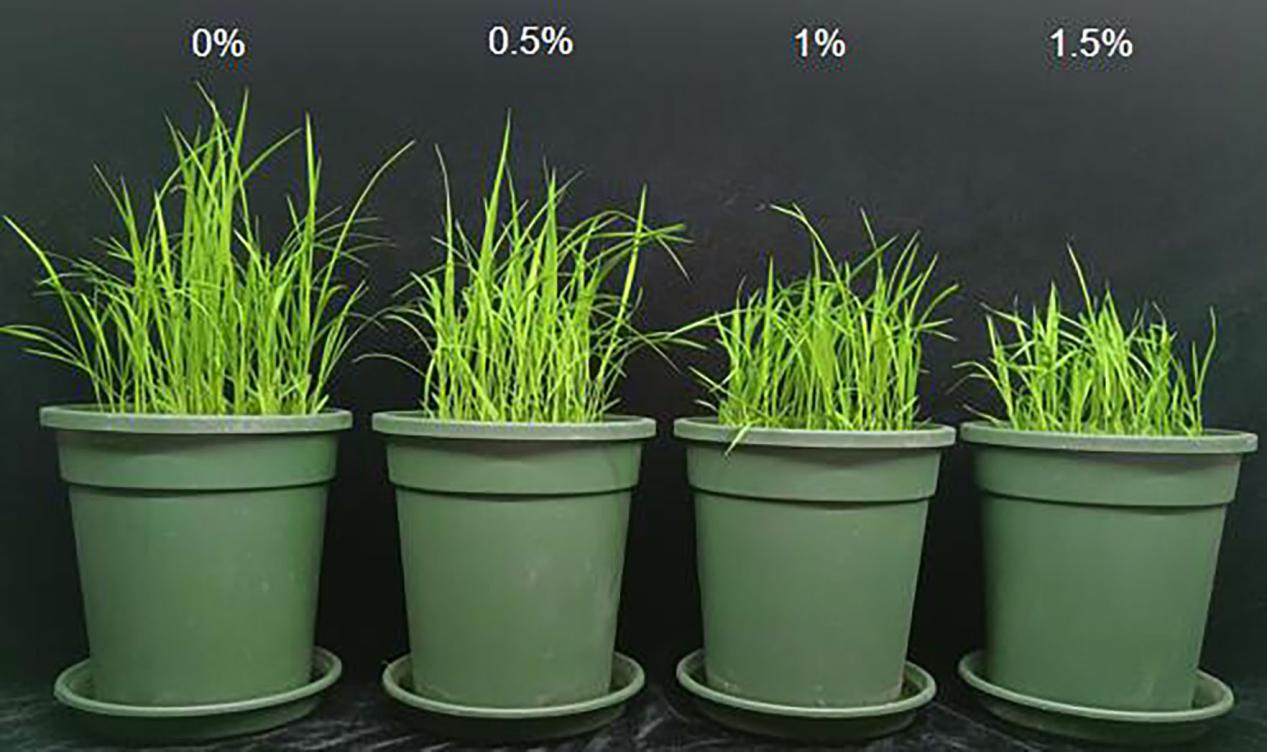
**

**Figure S1b**

**
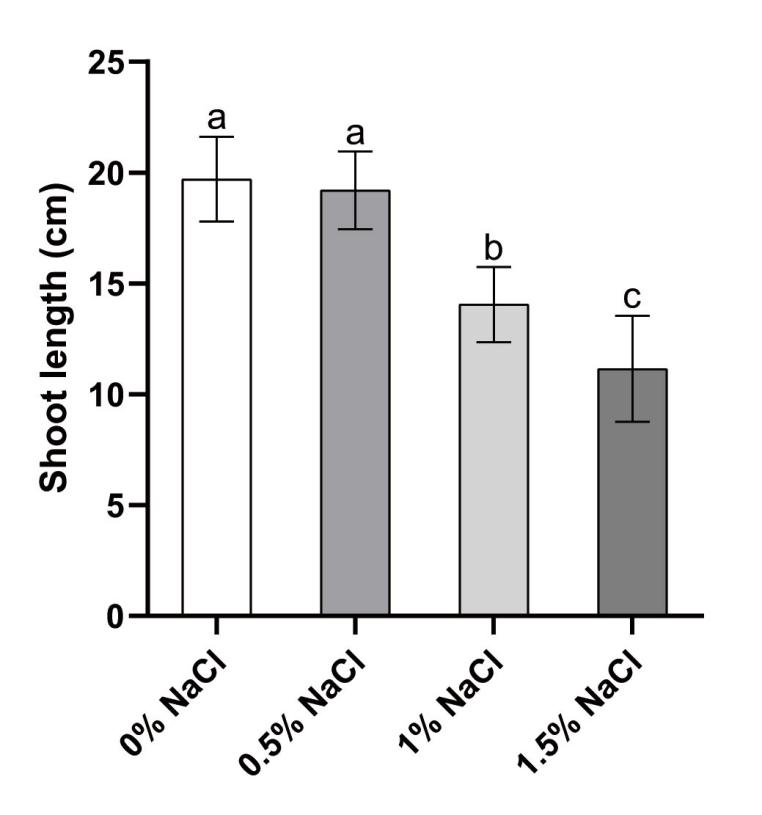
**

**Figure S2**

**
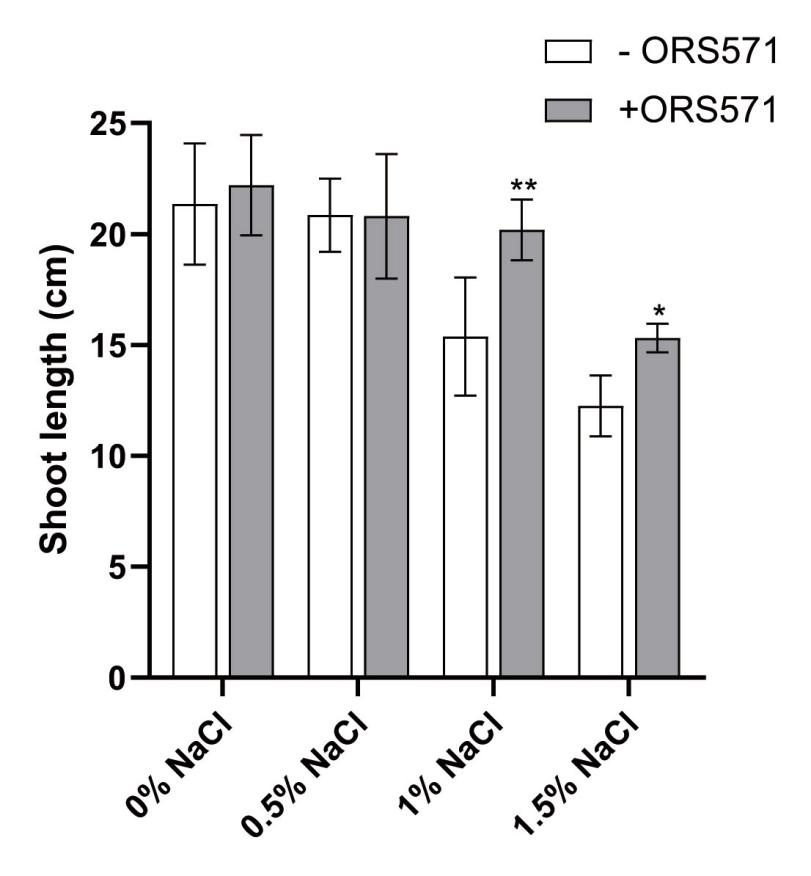
**
